# Supplementary material for: Compounding inequalities: Adolescent psychosocial wellbeing and resilience among refugee and host communities in Jordan during the COVID-19 pandemic
Source: PLoS One. 2022 Feb 2;17(2):e0261773. doi: 10.1371/journal.pone.0261773 (PMC8809558; doi:10.1371/journal.pone.0261773)
Supplement: S1 Table — (DOCX) [file pone.0261773.s001.docx]

| Table S1: Determinants of being surveyed at COVID-R1 or/and COVID-R2 | | | | |  |
| --- | --- | --- | --- | --- | --- |
|  |  |  |  |  |  |
|  | Dependent Variable: =1 if surveyed in COVID-R1 or COVID-R2 | | Dependent Variable: =1 if Surveyed in COVID-R1 and COVID-R2 | |  |
| *Baseline variables* | Coefficient (s.e.) | | | |  |
|  | All | Syrians | All | Syrians |  |
| Asset Decile | 0.000 | 0.002 | -0.004 | -0.001 |  |
|  | (0.002) | (0.003) | (0.003) | (0.003) |  |
| =1 if female | -0.006 | -0.022 | 0.031 | 0.009 |  |
|  | (0.013) | (0.015) | (0.016) | (0.018) |  |
| =1 if young cohort (10-12 at baseline) | 0.022 | 0.025 | 0.094 | 0.107 |  |
|  | (0.023) | (0.025) | (0.028)** | (0.032)** |  |
| =1 if enrolled in school | 0.006 | -0.009 | 0.012 | -0.009 |  |
|  | (0.023) | (0.025) | (0.028) | (0.030) |  |
| Highest grade attended | -0.002 | -0.001 | 0.003 | 0.009 |  |
|  | (0.004) | (0.004) | (0.005) | (0.005) |  |
| =1 if ever married | 0.012 | 0.039 | -0.004 | 0.052 |  |
|  | (0.033) | (0.035) | (0.040) | (0.043) |  |
| =1 if has disability | -0.008 | -0.008 | -0.010 | -0.003 |  |
|  | (0.017) | (0.019) | (0.020) | (0.023) |  |
| =1 if adolescent Syrian | 0.015 |  | 0.035 |  |  |
|  | (0.024) |  | (0.029) |  |  |
| =1 if adolescent Jordanian | 0.007 |  | -0.097 |  |  |
|  | (0.028) |  | (0.034)** |  |  |
| =1 if live in informal tented settlement |  | 0.048 |  | 0.097 |  |
|  |  | (0.025) |  | (0.032)** |  |
| =1 if live in camp |  | 0.010 |  | 0.000 |  |
|  |  | (0.016) |  | (0.019) |  |
| Mean of the outcome | 0.823 | 0.826 | 0.640 | 0.663 |  |
| Sample Size | 4,025 | | | |  |
| *Notes:* Table presents findings from a linear probability model where the dependent variable takes on a value of 1 if the adolescent is surveyed, and is 0 otherwise. * indicated p<0.05, ** p<0.01, and *** p<0.001 | | | | |  |
|  |  |  |  |  |  |
|  |  |  |  |  |  |
