# Supplementary material for: Compounding inequalities: Adolescent psychosocial wellbeing and resilience among refugee and host communities in Jordan during the COVID-19 pandemic
Source: PLoS One. 2022 Feb 2;17(2):e0261773. doi: 10.1371/journal.pone.0261773 (PMC8809558; doi:10.1371/journal.pone.0261773)
Supplement: S1 File — (DOCX) [file pone.0261773.s002.docx]

| S1 File: Description of GAGE COVID-R1 and COVID-R2 survey outcomes | | |
| --- | --- | --- |
| Outcome | Respondent/ Subgroup | Description of Outcome |
| Disruption to services (Table 4) | | |
| Household lost any employment due to pandemic | Adult female caregiver* | Indicates whether any household member lost employment (including self-employment) due to the pandemic and subsequent control measures, either temporarily or permanently. Note that households where no one was working prior to the pandemic are considered to not have lost any employment. |
| Household lost any income due to pandemic | Adult female caregiver* | Indicates whether the household lost any income due to the pandemic, where answer options include “all,” “most,” “some,” or “none.” |
| Household unable to buy essential food items in last week | Adult female caregiver* | Adapted from the Yale COVID-19 survey [1], this outcome indicates whether the respondent was able to buy all essential food items needed in the last 7 days prior to the survey. |
| Household severely food insecure (FIAS) | Adult female caregiver* | This indicator relies on the Food Insecurity Access Scale (FIAS), which asks a series of questions about household food insecurity during the last 4 weeks [2]. A shortened version of the scale used in the GAGE COVID-19 survey uses 3 questions on food insecurity experiences—each followed by a question about frequency of the experience. The shortened FIAS is scored on a scale of 0 – 9, where a score of 1 or higher indicates severe food insecurity. |
| Adolescent unable to access healthcare due to pandemic | Adolescent respondents who reported needing healthcare | Adapted from the EMERGE COVID-19 and Gender Survey Questions instrument [3], this indicator identifies the adolescents who reported needing to see a doctor or healthcare provider or to obtain medication at any point since the pandemic began (March 2020), but were *not* able to do so due because of pandemic-related obstacles including, for example, government restrictions on travel and mobility, closure of or restrictions on access to healthcare facilities, fear of becoming sick with COVID-19, or fear of being perceived as sick with COVID-19 by visiting a healthcare facility, among other reasons. |
| Adolescent self-reported hunger in last 4 weeks | Adolescent respondent | This measure captures the adolescent’s response to the following statement: *“Now I am going to read you a statement that some young people have made about their food situation. I feel hungry, because there is not enough food to eat. In the last four weeks, this has happened…”*, where answer options include “many times/more than 2 times,” “1 or 2 times” and “never.” |
| Psychosocial Wellbeing (Table 5) | | |
| PHQ-8 results indicate moderate-severe depression (≥ 10) | Adolescent respondent | The Patient Health Questionnaire 8 (PHQ-8) is a brief screening questionnaire for depression [4], where the respondent answers a series of 8 questions around mental health symptoms in the past two weeks. The questionnaire is scored on a scale of 0-24, where a score of greater than or equal to 5 indicates mild depression, 10 indicates moderate depression, 15 indicates moderately severe, and 20 indicates severe depression. For this analysis, this outcome is summarized as a binary indicator for the adolescent scoring 10 or higher on the screening, indicating moderate to severe depression. |
| GAD-7 results indicate for moderate-severe anxiety (≥ 10) | Adolescent respondent | The General Anxiety Disorder 7 (GAD-7) is a validated screening tool for Generalized Anxiety Disorder [5], and has also demonstrated validity for other anxiety disorders including Panic Disorder, Social Anxiety, and Posttraumatic Stress Disorder. The questionnaire is scored on a scale of 0 to 21, where a score of greater than or equal to 5 indicates mild anxiety, greater than or equal to 10 indicates moderate anxiety, and greater than or equal to 15 indicates severe anxiety. For this analysis, this outcome is summarized as a binary indicator for the adolescent scoring 10 or higher on the screener, indicating moderate to severe anxiety. |
| Adolescent believes people in community are more anxious/depressed | Adolescent respondent, age 15 and older | Adapted from the EMERGE COVID-19 and Gender Survey Questions instrument [3], the GAGE survey includes a series of questions on perceived changes in the community as a result of the COVID-19 pandemic and efforts to control the virus. This outcome indicates that the adolescent responded “yes” to the statement “More people are becoming very anxious or depressed.” |
| Adolescent thinks there is an increase in thoughts of self-harm in community | Adolescent respondent, age 15 and older | As the above, this outcome is adapted from the EMERGE COVID-19 and Gender Survey Questions instrument question series on impacts of the pandemic in the community [3]. This outcome indicates that the adolescent responded “yes” to the statement “There is an increase in thoughts about self-harm, or people harming themselves.” |
| Adolescent is fearful or embarrassed to ask family for menstrual hygiene management support | Female adolescent respondent who reported reaching menarche | Adapted from the EMERGE COVID-19 and Gender Survey Questions instrument [3], the GAGE survey includes a series of questions asked to female adolescents around menstrual hygiene management. This outcome indicates that the female adolescent respondent who has begun menstruation agrees or partially agrees with one or more of the statements “I am embarrassed about asking family members to support me with my menstrual management” and “I am fearful about asking family members to support me with my menstrual management.” |
| Adolescent worries about marrying earlier due to pandemic | Adolescents who have never been married | Indicates that the adolescent agrees or partially agrees with the statement “I worry I will marry earlier as a result of the Corona situation.” Note that this question is only asked to adolescents who have never been married. |
| Adolescent agrees pressure to marry decreased due to pandemic | Adolescents who have never been married | Indicates that the adolescent agrees or partially agrees with the statement “The Corona situation has decreased the pressure on me to get married.” Note that this question is only asked to adolescents who have never been married. |
| Coping Strategies (Table 6) | | |
| Low Resilient Coping Score (0-9), COVID-R2 only | Adolescent respondent | Adapted from the EMERGE COVID-19 and Gender Survey Questions instrument [3], the GAGE COVID-R2 survey administered the Brief Resilient Coping Scale (BRCS) to all adolescents. The BRCS consists of 4 questions on the individual’s behavior and actions surrounding coping in difficult situations and an individual’s ability or tendency to cope with stress adaptively [6], where answer options range from 0 “does not describe me at all” to 4 “describes me very well”—although some iterations of the BRCS use a scale of 1-5, with the same answer options. The BRCS is scored on a scale of 0-16, where higher scores indicate higher resilient coping. This outcome identifies adolescents who scored within range for low resilient coping tendencies, with a score of 0-9. |
| High Resilient Coping Score (13-16), COVID-R2 only | Adolescent respondent | Based on the BRCS (described above), this indicator identifies adolescents who scored within range for high resilient coping tendencies, with a score of 13-16. |
| Adolescent reports coping well with stress caused by pandemic, COVID-R2 only | Adolescent respondent | Following the BRCS, the GAGE COVID-R2 survey asks an additional 4 questions in the same format as the original BRCS scale focused specifically on coping during the COVID-19 pandemic, including this outcome. This outcome identifies adolescents who agreed that the statement “I am coping well with the difficulty and stress caused by the Corona/COVID-19 pandemic” either “describes me” or “describes me very well.” |
| Adolescent reports seeking comfort and guidance from religion, COVID-R2 only | Adolescent respondent | Following the BRCS, the GAGE COVID-R2 survey asks an additional 4 questions in the same format as the original BRCS scale focused specifically on coping during the COVID-19 pandemic, including this outcome. This outcome identifies adolescents who agreed that the statement “I seek comfort and guidance from religion, such as by praying, reading from the holy book, or other religious activities” either “describes me” or “describes me very well.” |
| Adolescent ever smoked cigarettes (males age 15+ only) | Male adolescent respondents, age 15 and older | Indicates that the adolescent responded “yes” to the question “Have you ever smoked cigarettes?” Note that this question is only asks to adolescents age 15 and older. Beginning at COVID-R2, this question was only asked to male adolescents. Throughout this paper, this outcome is presented among adolescent males age 15 and older only. |
| Increased smoking in pandemic (among regular smokers, males age 15+ only) | Male adolescent respondents, age 15 and older | Indicates that the adolescent responded to the question “Compared to the time right before the Corona situation to now, are you smoking more, less, or about the same number of cigarettes as usual?” with “More.” Note that this outcome excludes all adolescents who have never smoked cigarettes, as well as those adolescents who reported not being regular smokers. |
| Decreased smoking in pandemic (among regular smokers, males 15+ only) | Male adolescent respondents, age 15 and older | Indicates that the adolescent responded to the question “Compared to the time right before the Corona situation to now, are you smoking more, less, or about the same number of cigarettes as usual?” with “Less.” Note that this outcome excludes all adolescents who have never smoked cigarettes, as well as those adolescents who reported not being regular smokers. |
| Social Connections (Table 7) | | |
| Adolescent received family support for schooling (among enrolled pre-pandemic) | Adolescent respondents who reported school enrollment pre-pandemic | Indicates that the adolescent responded “Yes” to the question “Is your family providing any support for learning while schools are closed”, regardless of the type of support provided. Note that this question is only asked to adolescents who 1) indicated that they were enrolled in school prior to the pandemic in March 2020 or 2) indicated that they re-enrolled in school in-person during Jordan’s brief school reopening in September 2020 (COVID-R2 only). |
| Household had contact with a teacher in last week (among enrolled pre-pandemic) | Adolescent respondents who reported school enrollment pre-pandemic | Indicates that the adolescent responded “Yes” to the question “Have you or someone in your household been in contact with a teacher in the last 7 days?” Note that this question is only asked to adolescents who 1) indicated that they were enrolled in school prior to the pandemic in March 2020 or 2) indicated that they re-enrolled in school in-person during Jordan’s brief school opening in September 2020 (COVID-R2 only). |
| Adolescent reports that household stress increased | Adolescent respondent | Indicates that the adolescent agrees or partially agrees with the statement “The Corona situation has increased the stress in my household.” |
| Adolescent has friend they can trust (COVID-R2 only) | Adolescent respondent | Indicates that the adolescent responded “Yes” to the question “Do you have any friends, who are not members of your household, that you trust and with whom you can talk about feelings and personal matters or call on for help?” |
| Adolescent interacted with friends or non-household family in person in last week | Adolescent respondent | Adapted from the Yale COVID-19 Survey and the Mindset COVID-19 survey [1, 7], this outcome indicates that the adolescent left the home—or had visitors to their home—in the past 7 days, with the purpose of socializing with friends or non-household family members. |
| Adolescent had no contact with friends in last week, in -person or virtually (COVID-R2 only) | Adolescent respondent | Adapted from the Save the Children International survey, “Hidden Impact of COVID-19 on Children” [8], this outcome indicates that the adolescent reported having no contact with their friends at all in the past 7 days, including hanging out or playing in person, talking/messaging on the phone, social media, and/or online games. |
| Adolescent has a personal mobile device with internet access | Adolescent respondent | Indicates that the adolescent has one or more personal mobile devices—including a mobile phone, tablet, or laptop—that they are able to use to access the internet. |
| Adolescent interacted with friends virtually in last week (COVID-R2 only) | Adolescent respondent | Adapted from the Save the Children International survey, “Hidden Impact of COVID-19 on Children” [8], this outcome indicates that the adolescent reported keeping in touch with their friends online or over the phone in the last 7 days, including talking or messaging on the phone, social media, and/or online games. |
| Adolescent self-reports increased technology access | Adolescent respondent | Adapted from the Mindset COVID-19 survey [7], this outcome indicates the adolescent responded to the question “Thinking about the current Corona situation/pandemic, how much do you feel that you have more access to technology (e.g., internet, phone) compared to before the pandemic?” with either “completely” or “a moderate amount” |
| Household currently receives any cash or food aid | Adult female caregiver* | Indicates that the household benefits from at least one of four large-scale cash or food aid programs in Jordan: UNHCR cash transfer programs (for refugee households); World Food Programme food vouchers (for Syrian refugee households); UNRWA food aid cards (for Palestinian refugee households); Hajati cash assistance (for vulnerable Jordanian and refugee households living in host communities and/or informal tent settlements). |
| Household cash or food aid decreased since pandemic began (among those households that receive any aid) | Adult female caregiver* | Indicates the adult survey respondent reports that the amount of food or cash provided through any of the four aid programs listed above decreased compared to aid immediately before the pandemic in March 2020. Note that if the household received more than one type of aid, this indicator identifies household that reported a decrease from any program, even if other types of aid did not decrease. |

*Note: In the event where there is no adult female caregiver or the adult female caregiver is not available, a shortened version of the survey is administered to another adult in the household. In the case of adolescents who live independently or are married, the shortened version of the survey may be administered to the adolescent him or herself.

**References**

1. Yale Research Initiative on Innovation and Scale (Y-RISE) and Innovations for Poverty Action (IPA). COVID-19 Survey for the Cox’s Bazaar Panel Survey Project. 2020. Available from: <https://yrise.yale.edu/covid-19-survey-for-the-coxs-bazaar-panel-survey-project/>

2. Coates J, Swindale A, Bilinsky P. Household Food Insecurity Access Scale (HFIAS) for measurement of household food access: indicator guide (v. 3). Washington, DC: Food and Nutrition Technical Assistance III Project (FANTA)/FHI 360; 2007. Available from: <https://www.fantaproject.org/monitoring-and-evaluation/household-food-insecurity-access-scale-hfias>

3. Center on Gender Equity and Health (GEH). EMERGE COVID-19 and Gender Survey Questions. 2020. Available from: <http://emerge.ucsd.edu/covid-19/>

4. Kroenke K, Strine TW, Spitzer RL, Williams JBW, Berry JT, Mokdad AH. The PHQ-8 as a measure of current depression in the general population. J Affect Disord. 2009; 114(1-3): 163-173. Available from: <https://pubmed.ncbi.nlm.nih.gov/18752852/>

5. Spitzer RL, Kroenke K, Williams JBW, Löwe B. A brief measure for assessing generalized anxiety disorder: the GAD-7. Arch Intern Med. 2006; 166(10): 1092-1097. Available from: <https://pubmed.ncbi.nlm.nih.gov/16717171/>

6. Sinclair VG, Wallston KA. The development and psychometric evaluation of the Brief Resilient Coping Scale. Assessment. 2004; 11(1): 94-101. Available from: <https://www.ncbi.nlm.nih.gov/pubmed/14994958>

7. Mindset. COVID-19 Survey. 2020.

8. Burgess, M, Sulaiman, M, Arlini, SM, Qaiser, MH, Thiyagarajah, S, Dulieu, N, et al. Hidden Impact of COVID-19 on Children: A Global Research Series. Save the Children International. 2020. Available from: <https://resourcecentre.savethechildren.net/library/hidden-impact-covid-19-children-global-research-series>
