# Supplementary material for: Compounding inequalities: Adolescent psychosocial wellbeing and resilience among refugee and host communities in Jordan during the COVID-19 pandemic
Source: PLoS One. 2022 Feb 2;17(2):e0261773. doi: 10.1371/journal.pone.0261773 (PMC8809558; doi:10.1371/journal.pone.0261773)
Supplement: Attachment — Submitted filename: PONE Paper Review_210904.pdf [file pone.0261773.s003.pdf]

Thank you for the opportunity to review the paper.

The study provides invaluable insights into an important social and global health challenge with the Impact of COVID-19 on vulnerable adolescents living in displaced/temporary residential communities in Jordan.

The authors present a mixed-method study using quantitative survey data collected in three rounds with a baseline round in 2018/2019 followed by two rounds of panel data collection in 2020 and 2021, where the effect of Covid induced lockdown on vulnerable adolescent's health and social development is presented. The survey data was collected as part of an ongoing research program titled 'Gender and Adolescence: Global Evidence (GAGE)' which follows close to 3000 adolescents living in camps and host communities in Jordan; while qualitative data were purposively collected from
Multivariate regression analysis is conducted on the pooled data comprising young and older adolescents of Syrian, Palestinian and Jordanian heritage on health and social development indicators drawn from the GAGE conceptual framework. The paper supplements quantitative findings with qualitative interviews from the sampled population and have creatively presented both findings. While the paper provides insights mainly from the effect of lockdown on the study population, the underlying pathways reflect the added challenges faced by a vulnerable community that are already disadvantaged due to geo-political and ongoing conflicts in the Middle East. The paper presents opportunities to advance public health interventions and policy targeting communities/adolescents living in vulnerable environments that may interest a wide readership.

The paper in its current form requires further revisions that are deemed to be minor, and most of these are provided to help improve the paper's readability and presentation of information. Currently, the paper combines quantitative and qualitative findings spread across eight tables, which seem disjoint in places. While the quantitative methodology is appropriate for the paper, the interlinkages with the qualitative section need to be further developed. Some of the comments below are the reviewer's reflections that are meant to encourage the authors of this impressive study

Introduction

Comment:

Introduction from pages 1-3 provides a general context of the impact of covid related lockdowns on adolescents health and wellbeing. The authors seek to imply the effect of educational disruptions and social isolation bought on by the lockdown as the primary pathway affecting wellbeing. While the first three introductory pages set the context, the research population for this paper is introduced much later on page 3 . Even outside of the pandemic, the health and wellbeing of vulnerable adolescents living in settlements camps and under settlements is already expected to be impacted due to disruption of the social determinants of health. The impact of the pandemic hence adds additional burden over and above an existing health risk. The paper currently does not adequately introduce the research population with relevant context. The authors on page 10 make reference to 'pre-existing social inequalities'; however, the preceding introduction does not substantiate what these are.

Comment: Page 10(line numbers-not provided in paper) As the pandemic now stretches >2 years; the authors may like to state the data collection dates earlier in relation to the following statement-three and nine months after its onset, on adolescent psychosocial wellbeing and mental health, exploring the role of support systems at the individual, household, community, and policy levels among communities affected by displacement

Methods.

Comment: The reference to dates (in pages 10,11)to explain the COVID-19 pandemic trajectory in Jordan was appreciated. However, as infection and case fatality rates are likely to be higher for vulnerable people living in camps where population density is higher, the authors may like to add a few references (if available about death/infection rates) in such communities which are different from the general population

Comment: Could you please define 'host communities' as its relevant to the study and not part of common knowledge?

Comment:References are missing on page 5, last para.- Drawing on the work of Amartya Sen [X] and Marta Nussbaum [X]

Comment: On page 16, table 3, panels a and b provide the paper's main quantitative findings across different timelines. While the effect sizes are provided, the authors may like to add a note on the underlying measure of association used to obtain the effect size(RR or OR, e.g.). Additionally, some of the measures reported currently don't have background information provided in the introduction(pages 1-3). This is a repeated comment as I have previously suggested adding few references(if available) in the introduction to provide readers with some context about the social vulnerabilities encountered by the study's population

Results Section:

Comment: The transition from quantitative results on page 27 to qualitative results on page 28 is linked well and allows a gradual transition to the qualitative findings under the section on 'COVID19-and adolescent psychosocial wellbeing'.

Comment: Some of the key characteristics in how ITS, camps and host families differ would provide valuable context to the paper. Adding details in the method or as a section in the introduction would be important. Page 28, last para reflects on the nature of social networks in ITS as a reason for better PHQ-8 scores. Readers would be better placed to link the insight with a previous description of characteristics as suggested.

Comment: While the paper has collected data from younger and older cohorts in baseline(pre-pandemic), the presentation of results in tables 3 and 4 and in subsequent sections is grouped around residential affiliation(camp versus ITS vs Host family). The qualitative insights presented on page 29 provides reflections using age as a reference which is valuable. I found that the quantitative section in table 5(page 31) presents age-based reflections. As the results move between qualitative and quantitative sections fairly, I would

advise the authors to have a summary section before or soon after the ‘results’ section starts to provide a pathway for readers to follow and improve the section’s linking.

Comment: The authors have previously mentioned on page 13 that the paper explores whether access to adolescent-specific programs in the form of ‘Makani centres act as a protective factor. This would be a valuable finding to report, especially in R1 and R2 of data collection if the centres were functional. I was unable to adequately tease out the effect of access to Makani centres on the outcomes produced in tables 3-5. I understand that not all residential camps may have the program, but if the authors have looked at it as one of the main interventions available in the camps, it would be valuable to have a section on it in results. This is especially important when reporting qualitative outcomes such as pages 28/39 where adolescents report higher mental health issues. This is relevant to readers understanding the association between availability of access to Makani centres on adolescent mental health(boys/girls and young vs old) during the data collection rounds.

Comment: The results on page 40 were insightfully presented, especially with regards to the associations of wealth and age on feeling pressured to marry early.

Comment: The limited evidence of illicit drug use, as reported on page 43, may also have been due to respondents withholding sensitive information. As qualitative interviews were done virtually, the opportunity to develop rapport between interviewee and interviewer is limited. However, the role of the social support offered by families may have a mitigating effect on illicit drug use. The influence of family support is evident on page 49 /table 7where family and community coping skills are presented.

Comment :The reduced aid offered to families during the pandemic, as explained on page 61 is an important determinant of household wellbeing and vulnerability and may explain some of the transmitted mental health stresses that are common within a household encountering common stressor(s)

The discussion and conclusion are well-presented sections
