# Supplementary material for: Compounding inequalities: Adolescent psychosocial wellbeing and resilience among refugee and host communities in Jordan during the COVID-19 pandemic
Source: PLoS One. 2022 Feb 2;17(2):e0261773. doi: 10.1371/journal.pone.0261773 (PMC8809558; doi:10.1371/journal.pone.0261773)
Supplement: Attachment — Submitted filename: Response to Reviewers Nov 23.docx [file pone.0261773.s004.docx]

November 23 2021

Dear Reviewers,

Many thanks for your additional review of our paper and to Reviewer 1 for confirming that all of their substantive comments were addressed. In response to your request for minor revisions, we would like to note the following:

i) Regarding the datasets, we have now submitted the Jordan Covid-19 data to the UK Data Archive and it has now been accepted (as of November 22 2021) and will be live within the next month based on standard timelines.
ii) We have also reviewed the references so that where there are multiple references we have used hyphens rather than listing all numbers sequentially. We have kept this in track changes to show the reviewer the actions taken.
iii) We have included the requested funding statement: "The funders had no role in study design, data collection and analysis, decision to publish, or preparation of the manuscript."

We hope that we have now addressed all outstanding Reviewer comments

Thanks and best wishes

Nicola Jones on behalf of the authors
